# Resistance mechanisms and translational bottlenecks in CAR-T cell therapy for pediatric acute lymphoblastic leukemia: a perspective

**DOI:** 10.3389/fimmu.2026.1909384

**Published:** 2026-10-06

**Authors:** JiLi Xu, Lili Sui, Wenzhe Yang, Li Xu, Mingliang Shan

**Affiliations:** 1 College of Traditional Chinese Medicine, Shandong Medical and Pharmaceutical University, Yantai, China; 2 Gaomi Maternity and Child Health Hospital, Gaomi, China; 3 Shandong University of Traditional Chinese Medicine, Jinan, China; 4 Shandong Second Medical University, Weifang, China

**Keywords:** allogeneic CAR-T, antigen escape, CAR-T cell therapy, chimeric antigen receptor, immunotherapy resistance, pediatric acute lymphoblastic leukemia, translational bottleneck

## Abstract

Chimeric antigen receptor (CAR)-T cell therapy has achieved remarkable remission rates exceeding 80% in relapsed/refractory pediatric B-cell acute lymphoblastic leukemia (B-ALL), yet 40–50% of patients eventually relapse within the first year. This therapeutic ceiling is not a single failure mode but the convergence of at least four interrelated layers: loss of CAR-T persistence, antigen escape driven by CD19 splicing variants and lineage switch, CAR-T cell-intrinsic exhaustion from chronic tonic signaling, and an immunosuppressive bone marrow microenvironment that actively extinguishes effector function. Our systematic analysis of 226 pediatric-eligible CAR-T trials shows that 95.6% are Phase I–II, 3.5% (8/226) are registered as Phase III/IV, and 58 of 102 completed trials (56.9%) had no outcome recorded in the source database. In this Perspective, we synthesize the molecular mechanisms of CAR-T resistance, contextualize them within the current clinical trial landscape, and critically evaluate emerging engineering strategies—from dual-targeting and armored CAR architectures to CRISPR-edited allogeneic products and *in vivo* mRNA delivery. We argue that progress depends on both smarter receptor design and structural reforms in trial design, outcome transparency and global collaboration.

## 1 Introduction

The approval of tisagenlecleucel in 2017 marked a transformative moment in pediatric oncology, offering a potentially curative option to children with relapsed or refractory B-ALL who had exhausted conventional therapies (1, 2). Complete remission rates of 80–89% in pivotal trials (3–5) established CAR-T therapy as a standard option for children with relapsed or refractory B-ALL, building on decades of progress that raised cure rates from zero in the 1950s to approximately 10% in the early 1960s and over 90% today (6), and the ensuing decade has witnessed an extraordinary expansion of clinical investigation: our search of the Trialtrove^®^ database identified 226 interventional CAR-T trials in pediatric-eligible ALL globally, with initiations accelerating since 2017.

Yet the narrative of unqualified success deserves careful scrutiny. The same trials that report striking initial responses also document 40–50% relapse rates within the first year, driven by four convergent resistance layers—loss of CAR-T persistence, antigen escape, T-cell exhaustion, and microenvironmental immunosuppression—that no single CAR design has fully addressed (7, 8). Furthermore, our analysis of the interventional pipeline reveals a stark imbalance: 95.6% of trials are concentrated in Phase I through II, and only 8 of 226 trials (3.5%) are currently registered as Phase III or IV, none of them exclusively pediatric. More troubling, 58 of 102 completed trials (56.9%) had no outcome recorded in the source database. This dissociation between early-phase innovation and late-phase activity warrants scrutiny rather than a verdict of biological or translational failure (Methods).

In this Perspective, we integrate these resistance mechanisms with trial-level evidence and then consider engineering strategies and structural priorities that we believe are essential for improving durable outcomes.

## 2 Methods

Data source and search strategy. Trialtrove^®^ (Informa Pharma Intelligence) was searched on 30 April 2026 using the query: (Drug Type is Biological > Cellular > Cell technology type > Chimaeric antigen receptor > CAR-T cell) AND (Patient Age Group is Children) AND (Study Design does not contain Observation) AND (Disease is Oncology: Leukemia, Acute Lymphocytic), returning 226 trials. The independent ClinicalTrials.gov API query (June 2026) returned 295 records under a broader scope; the 69-record difference reflects scope and eligibility criteria rather than validation. The two queries differ in scope and taxonomy: the curated cohort requires a CAR-T cell product, an ALL classification and age criteria permitting children; the independent query applies none of these filters. Of the 226 curated trials, 197 (87.2%) carry ClinicalTrials.gov identifiers, of which 128 were retrieved by the independent query, the remainder using product-code intervention names or broader condition terms (**Supplementary Tables S1-s1, Table S1-s2**); an updated run of the independent query on 18 September 2026 returned 402 records, of which 222 matched a CAR-T intervention with an ALL condition by text and 149 of those permitted children; no additional records were added to the cohort; of the records retrieved by the independent query and registered on or before the search date, 34 ALL-specific CAR-T trials by registered text were absent from the cohort; the cohort is therefore presented as a defined source-based set, and completeness is a stated limitation.

Eligibility and definitions. Eligibility required an interventional CAR-T trial for ALL explicitly permitting children (<18 years); all 226 met these criteria (24 children-only). Records were de-duplicated and dual-reviewed; identifiers are in **Supplementary Table S1-s1**. “Disclosed outcome” = registry results field or linked publication (58/102 had none). Primary target hierarchical; multivalent products were counted once per contained antigen; durations n = 42; outcome direction (positive/negative/indeterminate) followed the recorded primary-endpoint status (met, not met, indeterminate). Registry-posted results, publications and conference reports were not distinguished, and recent completions may not yet have reported. Two records with non-standard phase labels were classified separately and are not counted as Phase III/IV. All 226 records retrieved by the query met these criteria, and no records were excluded at the screening step. Multi-country trials are counted in each country. The recorded figure (58/102, 56.9%) therefore indicates the absence of a recorded outcome rather than verified non-disclosure; a PubMed registry-identifier ([SI]) search of the 56 trials carrying ClinicalTrials.gov numbers identified a registry-indexed record for 15 (26.8%), any record for 16 (28.6%), and no record for 40 (71.4%) (**Supplementary Tables S1-s2**).

PubMed counts (**Figure 1M**): (Acute Lymphoblastic Leukemia) AND (CAR-T), June 2026.

**Figure 1:**
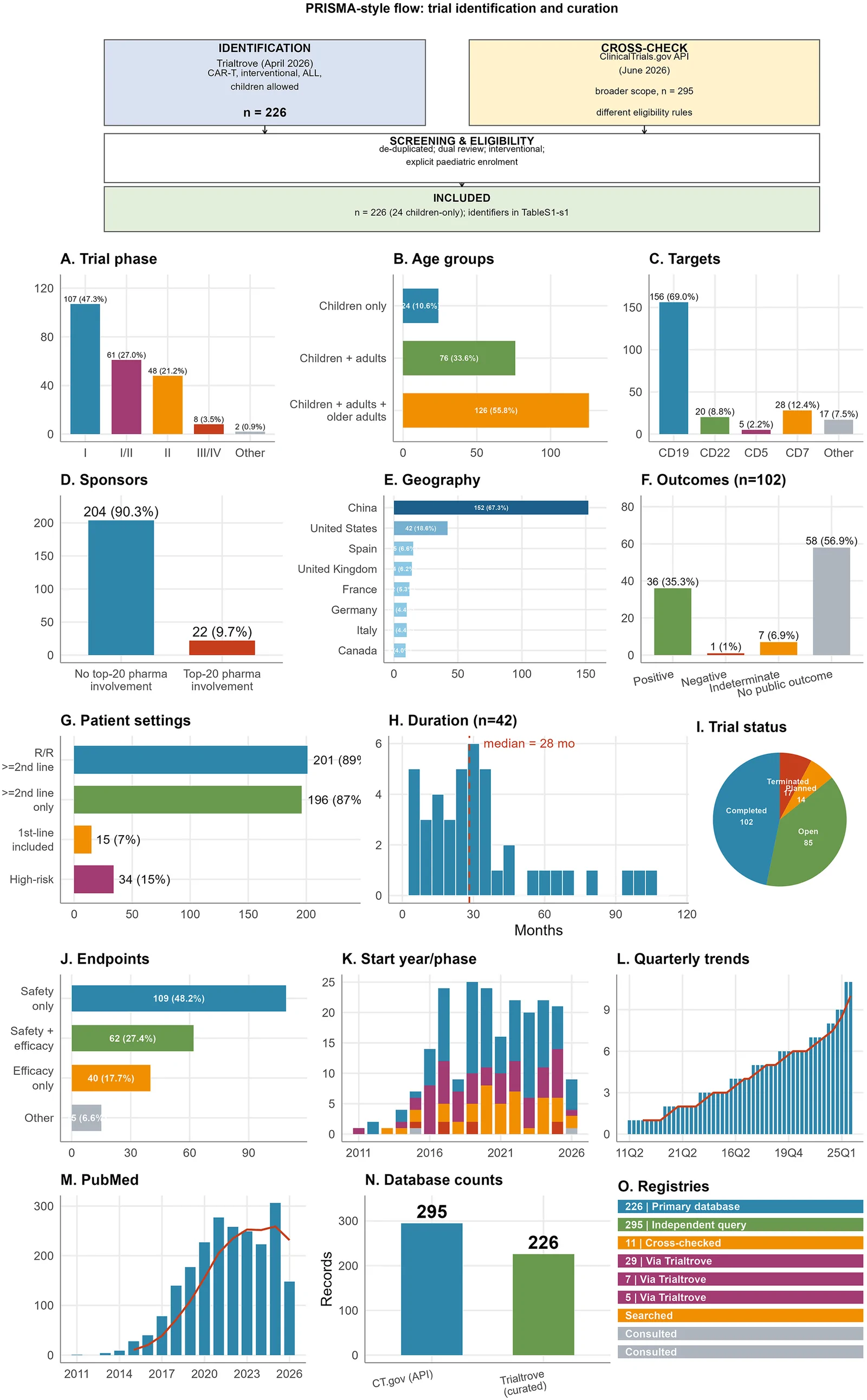
The 226 pediatric-eligible CAR-T trials for ALL: identification flow, trial characteristics, outcome transparency and dynamics. Upper panel: PRISMA-style flow diagram of trial identification and curation (Trialtrove^®^, April 2026; independent ClinicalTrials.gov cross-check, June 2026; see Methods). Lower panels: **(A)** Trial phase. **(B)** Registered age eligibility: children only (n = 24), children + adults (n = 76), children + adults + older adults (n = 126). **(C)** Primary molecular target (mutually exclusive). **(D)** Sponsor/collaborator involvement (top-20 pharma). **(E)** Geographic distribution (multi-country trials counted in each country). **(F)** Outcome disclosure among 102 completed trials (positive n = 36; negative n = 1; indeterminate n = 7; no public outcome n = 58). **(G)** Disease/setting focus (relapsed/refractory ≥ second line n = 201, of which 196 without first-line indication; high-risk n = 34; non-exclusive tags). **(H)** Reported enrollment duration (n = 42; red dashed line, median 28 months). **(I)** Trial status. **(J)** Primary endpoint categories. **(K)** Trial initiations by year and phase (2011–2026). **(L)** Quarterly initiation trends with 4-quarter moving average. **(M)** PubMed-indexed publication trends for (Acute Lymphoblastic Leukemia) AND (CAR-T). **(N)** Trialtrove curated cohort (n = 226) versus the broader ClinicalTrials.gov API query (n = 295); scopes differ, see Methods. **(O)** Registry coverage and cross-checks.

## 3 Molecular mechanisms of CAR-T resistance in pediatric ALL

The problem of resistance is best understood as a multi-layered barrier: it begins with failure of CAR-T persistence—the dominant CD19-positive relapse pattern—and is followed by antigen-dependent escape, exhaustion, and microenvironmental suppression; each layer must be addressed for durable responses.

### 3.1 Antigen escape, lineage plasticity, and target heterogeneity

Antigen escape is the most clinically well-characterized resistance mechanism in CD19 CAR-T therapy. CD19-negative relapse accounts for approximately 20–30% of relapses (roughly 10–25% of all treated patients) and arises through three principal mechanisms: alternative splicing of CD19 pre-mRNA (particularly exon 2 skipping yielding a truncated, non-functional protein isoform that evades CAR recognition), acquired frameshift and nonsense mutations in the CD19 gene that abrogate surface expression, and the most dramatic mechanism—lineage switch, in which B-ALL blasts transdifferentiate into a myeloid phenotype under the selective pressure of CD19 targeting (9, 10). This phenomenon is not merely a laboratory curiosity; it represents a fundamental limitation of single-antigen targeting in a disease defined by genetic plasticity.

Monovalent CD19 targeting remains vulnerable for three reasons: most relapses are CD19-positive and reflect poor CAR-T persistence (~70–80% of pooled pediatric relapses) (11, 12); target expression is quantitative—low CD19 density (13), trogocytosis (14) and rare cis-masking by transduced blasts (15) all lower it; and lineage switch, although rare, is enriched in KMT2A-rearranged/infant B-ALL (3/5 non-responders among 14 PRWCC infants), with prior blinatumomab adding risk (12, 16).

The field’s response has been the development of multi-targeted CAR architectures. CD22 has emerged as the most clinically advanced alternative target, with CD22-directed CAR-T cells demonstrating robust activity in CD19-negative relapse (17). In a single-arm Phase II study, sequential CD19- and CD22-directed CAR-T cells produced a 3-month objective response in 60 of 62 patients (97%) who received the target dose (18), and tandem CD19/CD22 bispecific constructs are now entering clinical evaluation. CD7-directed approaches are also gaining traction for T-ALL, where 28 trials (12.4% of the pipeline) are now active. In our view, the trajectory is clear: monovalent targeting will give way to multi-antigen recognition as the standard, though optimal stoichiometry, spacer design, and costimulatory domain combinations remain to be determined empirically.

### 3.2 CAR-T cell-intrinsic exhaustion

Even when antigen is present, CAR-T cells frequently lose effector function over time through a process of progressive exhaustion that mirrors, but is distinct from, physiological T-cell exhaustion. Chronic tonic signaling—antigen-independent clustering of CAR scFv domains on the T-cell surface—drives sustained activation of NFAT and other exhaustion-associated transcription factors, leading to hierarchical upregulation of inhibitory receptors (PD-1, TIM-3, LAG-3, CTLA-4) and progressive loss of proliferative capacity, cytokine secretion, and cytolytic activity (19, 20). The choice of costimulatory domain critically modulates this trajectory: 4-1BB-based CARs have generally been associated with greater persistence and reduced exhaustion compared with CD28-based designs, although the evidence is largely preclinical (21) and head-to-head clinical comparisons are lacking and results vary; the 4-1BB-containing tisagenlecleucel achieves durable remissions in pediatric ALL (1, 5). These comparisons also depend on CAR design, manufacturing, disease and treatment context.

From our perspective, the exhaustion problem is fundamentally a product quality problem. Pediatric patients who have undergone multiple rounds of chemotherapy harbor pre-existing T-cell dysfunction—characterized by skewed terminal differentiation, shortened telomeres, and diminished proliferative reserve—that compromises the fitness of the starting apheresis material (22). Product composition—including the CD4+:CD8+ ratio—further influences CAR-T cell expansion, persistence, and clinical outcomes (23). This observation strongly supports allogeneic approaches that source T cells from healthy donors, an issue we return to in the Engineering section below.

### 3.3 The immunosuppressive bone marrow microenvironment

The bone marrow is not a passive scaffold but an active immunological sanctuary that suppresses CAR-T function through multiple convergent mechanisms. Mesenchymal stromal cells (MSCs) secrete TGF-β, IL-10, and indoleamine 2,3-dioxygenase (IDO), each of which independently impairs CAR-T proliferation and effector function (24). CXCL12–CXCR4-mediated niche retention physically limits CAR-T trafficking into leukemic niches, while myeloid-derived suppressor cells (MDSCs) and regulatory T cells (Tregs) further amplify the suppressive milieu (25). Importantly, these microenvironmental mechanisms do not operate in isolation: they synergize with antigen escape and T-cell exhaustion to create a multi-layered barrier that single-modality interventions are unlikely to overcome.

## 4 The translational landscape: what the trial pipeline reveals

Our systematic analysis of 226 interventional CAR-T trials whose registered age criteria explicitly permit pediatric enrollment provides a quantitative backdrop for assessing the field’s translational trajectory (**Figure 1**; **Supplementary Table S1**; the identifiers of all 226 trials are listed in **Supplementary Table S1-s1**). The dataset was curated from Trialtrove^®^ and cross-checked as detailed in Methods (**Figures 1N, O**; **Supplementary Table S1-s2**). The analysis extends prior observations (11).

First, the pipeline is overwhelmingly early-phase: 107 trials (47.3%) are Phase I, 61 (27.0%) are Phase I/II, and 48 (21.2%) are Phase II, together representing 95.6% of all interventional activity (**Figure 1A**; **Supplementary Table S1-s3**; target accrual, **Supplementary Table S1-s4**). Age distribution and age group by phase are detailed in **Figure 1B** and **Supplementary Table S1-s5**. Only 8 trials (3.5%) are currently registered as Phase III/IV. Trial status and initiations by year and phase are in **Figures 1I, K**. Because phase labels cannot measure maturation over time, we also examined the 87 trials initiated ≥6 years before the search date (≤2019): 5 (5.7%) are Phase III or IV and none of the 24 pediatric-exclusive trials is beyond Phase II. Tisagenlecleucel was approved for r/r pediatric ALL from the single-arm Phase II ELIANA study (1), so randomized Phase III trials are often neither feasible nor required in this population; phase labels reflect design and registration intent, not progression; limited Phase III/IV representation should not be equated with registrational activity, and we interpret it cautiously rather than as proof of translational failure (Methods). The problem is not a lack of activity: 17 trials were launched in the most recent four quarters (**Figure 1L**), with a parallel rise in PubMed publications (**Figure 1M**; **Supplementary Table S1-s6**). Because 2026 is a partial year, recent annual/quarterly counts should be read cautiously. The bottleneck lies downstream—from early-stage innovation to regulatory-grade evidence.

Second, outcome transparency is poor. Among 102 completed trials, 58 (56.9%) had no outcome recorded in the source database (**Figure 1F**), where a recorded outcome was defined as a registry results field or a linked publication in the trial outcome field (Methods; **Supplementary Table S1-s2**). While 36 trials (35.3%) reported positive outcomes, 7 (6.9%) were indeterminate, and 1 (1.0%) reported negative outcomes, the absence of any recorded outcome for the majority of completed studies remains a structural transparency problem, and we are cautious about inferring publication bias from such a field. The gap was not uniform: among the 74 completed trials with China as the first-listed country, 47 (63.5%) lacked disclosed outcomes, versus 11/28 (39.3%) elsewhere, and registries such as ChiCTR have no mandatory results field (Methods; **Supplementary Table S1-s1**). We argue that mandatory prospective registration and results reporting—enforced by journals, funders, and regulatory agencies—should be a priority for the field.

Third, the geographic and sponsorship landscape reveals structural vulnerabilities. China accounts for 152 trials (67.3%), followed by the United States (42; 18.6%), while European and other regions contribute modestly (**Figure 1E**; **Supplementary Table S1-s7**); trials conducted in more than one country are counted in each country (Methods). This concentration raises questions about generalizability: 204 trials (90.3%) had no top-20 pharmaceutical company among sponsors or collaborators (**Figure 1D**; **Supplementary Table S1-s1**). Absence of a top-20 sponsor or collaborator is not equated with academic sponsorship (other industry, biotechnology, hospital and government entities may be present), and late-phase capacity cannot be inferred from phase and sponsor data alone. The median reported enrollment duration was 28 months (IQR 17–41; n = 42 trials with available duration data; **Figure 1H**; **Supplementary Table S1-s8**), further underscoring the heterogeneity of trial execution.

Finally, the target landscape, while diversifying, remains heavily concentrated by primary target: CD19-directed approaches account for 156 trials (69.0%), with CD22 (20; 8.8%) and CD7 (28; 12.4%) as the leading alternatives (**Figure 1C**; **Supplementary Table S1-s9**); when all antigens in combination products were counted, 54 trials (23.9%) included CD22, while the CD7 total was unchanged at 28 (12.4%) (Methods). The predominance of safety endpoints (109 trials; 48.2%) reflects the early-phase composition of the pipeline (**Figure 1J**), and most trials addressed relapsed/refractory (≥ second-line) settings (201; 89.0%), 196 (86.7%) exclusively so, with 34 (15.0%) high-risk-focused (**Figure 1G**).

## 5 Engineering the next generation: strategies to overcome resistance

The convergence of biological understanding and translational mapping provides a rational framework for evaluating emerging engineering solutions. We focus here on three approaches that we believe hold the greatest promise for addressing the resistance mechanisms outlined above: multi-targeted receptor design, allogeneic platforms, and *in vivo* CAR generation.

### 5.1 Multi-targeted and logic-gated CAR architectures

Dual-targeting strategies represent the most immediate response to antigen escape. Tandem CD19/CD22 CAR constructs, in which two scFv domains are linked in a single receptor, have shown promising preclinical and early clinical activity (26). An alternative approach—sequential infusion of monospecific CAR-T products—has already achieved clinical validation (18). Looking forward, we anticipate that logic-gated receptors (e.g., AND-gate CARs requiring dual antigen recognition for full activation, or NOT-gate CARs incorporating inhibitory modules to protect normal tissues) will enable more precise tumor discrimination. The field is moving, in our view, from “target any single antigen” toward “recognize the antigen combination that defines the tumor”.

### 5.2 Armored CARs and microenvironment remodeling

The recognition that CAR-T cells fail not only because of antigen loss or exhaustion but also because the bone marrow microenvironment actively suppresses them has motivated the development of “armored” fourth-generation CARs (TRUCKs) that secrete immunostimulatory cytokines such as IL-12, IL-18, or IL-15 upon CAR engagement (27). These payloads are designed to remodel the tumor microenvironment by recruiting innate immune effectors, counteracting TGF-β-mediated suppression, and supporting CAR-T persistence. While early-phase trials are ongoing, we caution that cytokine-secreting CARs introduce new risks—including systemic cytokine toxicity and unregulated inflammatory responses—that will require careful clinical monitoring and, potentially, safety-switch mechanisms.

### 5.3 CRISPR-engineered allogeneic CAR-T cells

Perhaps the most transformative development on the horizon is the shift from autologous to allogeneic CAR-T platforms. The rationale is compelling: allogeneic products derived from healthy donor T cells eliminate manufacturing delays, avoid the functional defects inherent in patient-derived cells, and enable off-the-shelf availability. Feasibility of genome-edited allogeneic CAR-T cells has been demonstrated in early clinical studies (28). Preclinically, CRISPR-Cas9 multiplex editing has been used to disrupt TRAC and B2M—distinct strategies against graft-versus-host disease and host-mediated rejection, respectively (29, 30). Persistence remains limited compared with autologous products, and reduced exhaustion is unproven in pediatric products, so such claims require caution. We view allogeneic platforms not as a replacement for autologous CAR-T but as a complementary strategy that will be particularly valuable for patients with rapidly progressive disease who cannot wait for personalized manufacturing.

### 5.4 *In vivo* CAR-T generation

The most radical departure from current practice is the generation of CAR-T cells entirely *in vivo*, using lipid nanoparticle (LNP)-encapsulated mRNA targeted to T cells via CD5-directed antibody conjugation (31). This approach bypasses ex vivo manufacturing entirely, eliminating the logistical complexity, cost, and quality variability that currently limit access to CAR-T therapy. *In vivo* CAR generation has been demonstrated preclinically in a cardiac injury model (31), and early-phase oncology studies have now begun: Phase 1 trials of *in vivo* CAR-T generation are ongoing, with CD19-directed (NCT06528301) and CD20-directed (NCT06539338) programs in relapsed/refractory B-cell malignancies and an *in vivo* BCMA-directed program in Phase 1 for multiple myeloma (NCT07075185). Delivery specificity, durability and safety remain unresolved. In our assessment, this approach faces substantial hurdles—including T-cell specificity, transfection efficiency in the bone marrow niche, and the inability to enrich for desired cell subsets—but its potential to democratize CAR-T therapy makes it one of the most important areas to watch.

## 6 Discussion

The central argument of this Perspective is that resistance to CAR-T therapy in pediatric ALL is not a single problem to be solved but a convergent failure mode at the intersection of tumor biology, T-cell physiology, and clinical development infrastructure. Antigen escape, T-cell exhaustion, and microenvironmental suppression each represent tractable targets individually; their combination, however, creates a barrier that demands coordinated multi-pronged strategies.

The pipeline analysis reveals a field that is simultaneously productive and constrained. Because phase labels cannot measure progression, we base this inference on the maturation cohort: among the 87 trials initiated ≥6 years before the search date, only 5 (5.7%) are Phase III/IV and none of the 24 pediatric-exclusive trials is beyond Phase II. Defined operationally (regulatory approval or registrational evidence), successful translation also remains limited: tisagenlecleucel is the only CAR-T product approved for pediatric r/r ALL (1). The absence of a recorded outcome for 58 of 102 completed trials further deprives the field of evidence for rational trial design (**Supplementary Table S1-s2**).

What, then, is to be done? We offer three priorities. First, the field urgently needs global harmonization of pediatric CAR-T trial design, including standardized endpoint definitions, mandatory long-term follow-up protocols, and prospective registration with results disclosure as a condition of ethical approval. Second, academic–industry consortia should be established to pool resources for late-phase trials, addressing the structural gap between investigator-driven early innovation and the infrastructure required for regulatory-grade evidence. Third, regulatory agencies should create specific incentives for pediatric cell therapy development, including expedited review pathways and conditional approval frameworks that recognize the unique ethical and practical challenges of pediatric oncology trials.

On the scientific front, the most promising advances will combine strategies in one product: a CRISPR-edited allogeneic tandem CD19/CD22 CAR-T cell with a cytokine payload and safety switch. Such a product would address all resistance layers; the challenge is clinical validation.

Finally, clinical and operational realities will determine success: CRS, ICANS, cytopenias, infections and hypogammaglobulinemia require structured care (immunoglobulin replacement) (1, 8, 25); consolidative HSCT was used in a subset of the sequential-therapy cohort (18); infant apheresis, dosing and vein-to-vein logistics motivate allogeneic platforms (26, 28); and rare secondary T-cell malignancies mandate long-term surveillance (32).

Manufacturing scalability, GMP capacity, product release, cost, regulatory complexity and healthcare-system capacity are also key translational bottlenecks; rapid or decentralized autologous manufacturing is being pursued alongside allogeneic and *in vivo* approaches to shorten vein-to-vein times (12, 26, 28).

In conclusion, the treatment of pediatric ALL with CAR-T therapy stands at a crossroads. The biological understanding of resistance has matured to the point where rational design of next-generation products is possible. The translational infrastructure to evaluate these products, however, lags behind. Closing this gap remains, in our view, the defining challenge for the field over the next decade.

## Data Availability

The original contributions presented in the study are included in the article/**Supplementary Material**. Further inquiries can be directed to the corresponding author.
